# Expression and Biological Evaluation of an Engineered Recombinant L-asparaginase Designed by *In Silico* Method Based on Sequence of the Enzyme from *Escherichia coli*

**DOI:** 10.34172/apb.2023.085

**Published:** 2023-06-12

**Authors:** Mahrokh Dastmalchi, Mahdiyeh Alizadeh, Omid Jamshidi-Kandjan, Hassan Rezazadeh, Maryam Hamzeh-Mivehroud, Mohammad M Farajollahi, Siavoush Dastmalchi

**Affiliations:** ^1^Department of Medical Biotechnology, Faculty of Allied Medical Sciences, Iran University of Medical Sciences, Tehran, Iran.; ^2^Infectious and Tropical Diseases Research Center, Tabriz University of Medical Sciences, Tabriz, Iran.; ^3^Biotechnology Research Center, Tabriz University of Medical Sciences, Tabriz, Iran.; ^4^Department of Pharmacology and Toxicology, School of Pharmacy, Tabriz University of Medical Sciences, Tabriz, Iran.; ^5^Pharmaceutical Analysis Research Center, Tabriz University of Medical Sciences, Tabriz, Iran.; ^6^Faculty of Pharmacy, Near East University, POBOX:99138, Nicosia, North Cyprus, Mersin 10, Turkey.

**Keywords:** L-asparaginase, Recombinant protein, Site-directed mutagenesis, Protein design

## Abstract

**Purpose:**

Medical usage of L-asparaginase (ASNase), the first-line of acute lymphoblastic leukemia treatment, is linked to allergic responses and toxicities, which necessitates the development of new bio-better ASNases. The aim of the current study was *in silico* design of a novel ASNase with predicted improved enzymatic properties using strategies encompassing sequence-function analysis of known ASNase mutants. Additionally, current study aimed to show that the new enzyme is active.

**Methods:**

Based on 21 experimentally reported mutations for ASNase, a virtual library of mutated enzymes with all 7546 possible combinations of up to 4 mutations was generated. Three-dimensional models of proposed mutant enzymes were built and their *in silico* stabilities were calculated. The most promising mutant was selected for preparing a genetic construct suitable for expression of the designed ASNase in bacterial cells.

**Results:**

Computational study predicted that Y176F/S241C double mutation of *Escherichia coli* ASNase may increase its folding stability. The designed ASNase was expressed in two different *E. coli* strains (Origami B(DE3) and BL21(DE3)pLysS) and then the soluble fractions prepared from the cell lysates of the host cells were used in enzyme activity assay. Results showed that enzyme activity of soluble fraction from Origami (95.4 ± 7.5 IU/0.1 mL) was four times higher than that of soluble fraction from pLysS (25.8 ± 2.5 IU/0.1 mL).

**Conclusion:**

A novel functional double mutant ASNase with predicted improved enzymatic properties was designed and produced in *E. coli.* The results of the current study suggest a great commercial potential for the identified enzyme in pharmaceutical and industrial applications.

## Introduction

 Acute lymphoblastic leukemia (ALL) is a bone marrow hematologic malignancy, which accounts for the most common type of cancer (25%) and disease related death in children.^1^ L-asparaginase (ASNase) was a breakthrough in the treatment of ALL with 93% of pediatric patients reaching complete remission after its administration.^2^ This therapeutic agent exerts its effect by catalyzing L-asparagine (Asn) hydrolysis to L-aspartic acid and ammonia. Malignant cells need Asn as a necessary extracellular nutrient, because, unlike normal cells, they are unable of synthesizing this amino acid. Depletion of Asn due to ASNase treatment eventually leads to starvation-induced apoptosis of cancerous cells.^3,4^ In addition to its indication in ALL, ASNase contributes to the treatment of several other hematological and non-hematological disorders including pancreatic cancer, lymphosarcoma, and acute myeloid leukemia. Not to mention its acrylamide (AA) mitigation properties in reducing AA formation during food processing.^5^ Despite its numerous efficiencies, ASNase application is associated with several restrictions. Although the bacterial enzyme is notable for its superior substrate specificity and a longer half-life in comparison with other types found in animals, plants, yeast, and fungi, its clinical administration is restricted by immune reaction and antibody production against foreign L-asparaginase.^6-9^ Moreover, thrombotic complications along with other side effects are among major restrictions.^10-13^ In addition, L-asparaginase should be injected frequently due to its rapid plasma clearance in order to maintain the desired therapeutic level.^14,15^ Therefore, there is a current need for innovative bio-superior ASNase. A bio-superior (or bio-better) is a new drug with improved properties which is designed based on an existing biopharmaceutical.^16-18^ These improved properties include affinity, selectivity, and stability against degradation. Several investigations have shown unique approaches to avoid certain side effects, such as replacing *Escherichia coli*-derived L-asparaginase with *Erwinia chrysanthemi*-derived L-asparaginase to prevent hypersensitivity responses. This suggests that enzymes from various origins have diverse antigenic reactivity.^19^

 Enzyme engineering is a young discipline aiming to develop useful proteins and has been gaining popularity as a result of its numerous applications in a variety of fields, particularly in industrial setups.^20,21^ Enzyme engineering can be regarded as the technique of customizing existing enzymes or creating new enzymes with unique and enhanced features. Enzymes are engineered for better performance in terms of catalytic activity, substrate specificity, enantioselectivity, thermodynamic stability, and solubility, just to name a few. Successful enzyme engineering requires a critical interpretation of an enzyme’s structure and function and is achieved by changing amino acid sequences as the result of genes modification.^20^ The two main enzyme engineering strategies are rational design^22^ and directed evolution.^23^ Rational design strategy relies on fundamental knowledge about structure and function of enzymes, whereas, directed evolution strategy operates based on introducing random sequence variations.^24^

 Recombinant proteins are synthesized by biological processes within the host cells, and are called ‘recombinant’ because the DNA encoding them has been recombined or created in a host cell that is typically of a different species from which they originated. In 1982, recombinant human insulin was the first recombinant protein administered in therapeutics, and since then biomedical biotechnology have witnessed substantial advances.^25^

 The aims of the present study are design, expression and biological evaluation of a new recombinant L-asparaginase enzyme engineered based on the sequence of commercial L-asparaginase from *E. coli*. In this work, using *in silico* methods, a virtual library of *E. coli* L-asparaginase mutants was generated and evaluated for folding stability. The most promising mutant enzyme was produced exogenously in *E. coli* (Origami B(DE3) and BL21(DE3)pLysS strains). The proposed novel L-asparaginase was active with very low glutaminase activity compared to commercial enzyme and can be considered as an engineered bio-better for clinical use after further functional and clinical evaluations.

## Materials and Methods

###  Chemicals

 All reagents were of analytical grade. Mouse anti-All His Tag (HRP Conjugated) antibody was obtained from BioLegend Company. Commercial *E. coli* L-asparaginase (Bionase^®^) was from ZYDUS Pharma Ltd (India). Tryptone, yeast extract, isopropyl-β-D-thio galactopyranoside (IPTG), Triton X-100, trypsin, agar, glycerol, phenylmethylsulfonyl fluoride (PMSF), N,N,N’,N’-tetramethyl ethylene diamine (TEMED), anhydrous D-glucose, and sodium azide (NaN3) were acquired from AppliChem (Darmstadt, Germany). YTA plasmid extraction maxi and mini kit were purchased from Yektatajhiz (Iran). Taq 2X Master Mix Red was from Ampliqon (Denmark). Kpn1 was from Fermentas (Russia). All solutions were prepared using ultrapure water obtained from Milli-Q^®^ Gradient water purification system (Millipore Corporation, Bradford, MA, USA). All utilized *E. coli *strains were from Novagen (Darmstadt, Germany). Amersham ECL western blotting detection reagent manufactured by Arsham (India).

###  Novel asparaginase gene design

 Protein sequence of *E. coli* L-asparaginase was retrieved from UniProtKB (https://www.uniprot.org/) database.^26^ Different variants of L-asparaginase produced by single and/or multiple mutations reported in the literature were collected. The protein sequence for *E. coli *L-asparaginase (accession number: P00805) and the corresponding gene sequence were downloaded from UniProt and NCBI (NCBI Reference Sequence: NC_000913.3, Gene ID: 947454), respectively. All reported mutations which were shown experimentally to have improving effects on stability, enzymatic activity, and other desired properties of *E. coli *L-asparaginase were listed. Using an in-house developed Linux shell script all possible combinations of up to four mutations were listed. Then, the mutation list was used to build three-dimensional structural models of the proposed mutant L-asparaginases for the evaluation of their folding stability by using FoldX (Version 4)^27,28^ program running on Linux operating system (CentOS 6.8). The mutants were sorted based on their folding potential energy and the promising mutant was selected for experimental evaluation. Toxic and allergenic features of proteins were predicted using ToxinPred^29,30^ and AlgPred 2.0^31^ online servers.

 The gene sequence for the selected L-asparaginase mutant was generated based on the gene sequence of wildtype enzyme from *E. coli* (the gene corresponding to the commercially available *E. coli* L-asparaginase) and inserted in pET22b( + ) expression vector. The construct was designed to have a 6 × HIS tag, a suitable signal sequence, and a cleavage site for factor Xa protease. The final genetic construct was ordered for synthesis via Pishgam Biotech Co, Tehran, Iran.

###  Expression of recombinant ASNase enzyme

 The synthesized DNA construct harboring the gene for the designed L-asparaginase was transformed into competent *E. coli* DH5α cells for amplification and extraction according to the standard protocol. Briefly, a microtube containing 100 µL of thawed competent cells was inoculated with 1 µL of the synthesized construct reconstituted in water, and the mixture was incubated for 30 minutes on ice. The bacterial suspension then was incubated at 42 ºC for 45 seconds and instantly cooled down for 2 min on ice. Then, the bacterial mixture was diluted by 500 µL of SOC (tryptone 2% (w/v), yeast extract 0.5% (w/v), NaCl 10 mM, KCl 2.5 mM, MgCl_2_ 10 mM, glucose 20 mM) media and was incubated at 37 ºC with vigorous shaking for 45 minutes. Then the appropriate volume of cell suspension (2, 18, and 50 µL) was plated on ampicillin (100 µg/mL) selective media and kept in incubator at 37 ºC overnight. The following day, the obtained well isolated single colonies were used to inoculate 10 mL of LB media and do the overnight incubation as usual. The presence of the gene of interest was confirmed by plasmid extraction and PCR reaction using forward (5ʹ-GCTAGTTATTGCTCAGCGG-3ʹ) and reverse (5ʹ-TAATACGACTCACTATAGGG-3ʹ) primers. The amplified construct was transformed into chemically competent *E. coli* cells suitable for protein expression. The transformed cells were plated on LB ampicillin plates and incubated at 37 °C overnight. Subsequent day, a 10 mL LB + ampicillin medium was inoculated by a single colony and incubated overnight at 37 °C and used as starter culture. The starter cultures were diluted at 1:100 in fresh LB medium (1% tryptone, 0.5% yeast extract, 0.5% NaCl) supplemented with ampicillin (100 μg/mL) and incubated at 37 °C with shaking at 180 rpm until OD_600_ of 0.6. Then, the expression was induced by adding IPTG (1mM) and cells were grown either at 37 °C for 4 hours, or overnight at 20 °C with shaking at 150 rpm. Centrifugation at 5000 *g* for 15 minutes was used to harvest bacterial cells. After removing the supernatant, the obtained cell pellet was resuspended in the lysis buffer (50 mM Tris-HCl pH 8, 100 mM NaCl, 1% Triton X-100, 1.4 mM protease inhibitor (PMSF), 0.1% β-mercaptoethanol, 10 µg/mL DNase and 0.1 mg/mL lysozyme). Three rounds of freeze-thaw steps were utilized by using liquid nitrogen to efficiently disrupt the cells. Furthermore, the lysate was sonicated 5 times on ice at 60% amplitude for 30 seconds with 30-second intervals. Bacterial debris was removed by centrifugation at 12 000 *g* for 20 minutes at 4 °C, and the soluble fraction was stored at -20 °C until use. Total protein content of soluble fraction was determined using MicroBCA protein assay kit according to manufacturer’s instruction (Thermo Scientific, Rockford, USA).

###  Western blotting

 Western blotting was carried out for detecting the expressed L-asparaginase. Samples were denatured in sample buffer (5X sample buffer, 50 % (v/v) glycerol, 0.3 M Tris (pH 6.8), 0.1% bromophenol blue) and were subjected to 12% SDS-PAGE. Subsequently, the resolved proteins were transferred onto PVDF (polyvinylidene fluoride) membrane in transfer buffer (0.025 M Tris, 0.192 M glycine, 20% (v/v) methanol, pH 8.3) at 250 mA for 1 hour. The membrane was blocked with buffer supplemented with 5% skim milk for 1 hour with shaking at room temperature. Then, the membrane was incubated with mouse anti-His tag HRP conjugated antibody (at 1:3000 dilution) in 5% skim milk buffer overnight at 4 °C with shaking. The membrane was subjected to 4 times of washing steps with TBS-T (tween 20 0.05% v/v) each for 5 minutes. Amersham ECL western blotting detection reagent was used to visualize the protein bands using manufacturer’s instruction.

###  L-asparaginase and L-glutaminase activity assay

 The enzyme activity was determined by measuring the released ammonia during L-asparagine (or L-glutamine) hydrolysis using Nessler reagent.^32^ A 2.2 mL volume of the reaction mixture consisting of L-asparagine (8.6 mM) in 23 mM Tris-HCl (pH 8.6) was equilibrated at 37 °C for 10 minutes. Then, the mixture was added 0.1 mL of enzyme solution (or sample form soluble fraction of cell lysate), immediately mixed by inversion and incubated at 37 °C for 30 minutes. Subsequently, the reaction was stopped by adding 0.1 mL of 1.5 M trichloroacetic acid (TCA). The blank solution was prepared by adding the enzyme solution to reaction mixture treated with TCA. After centrifugation of the reaction mixture, 0.2 mL of clarified supernatant was mixed with 4.3 mL of ultrapure water and 0.5 mL of Nessler reagent (0.09 mol/L solution of potassium tetraiodomercurate(II) in 2.5 mol/L potassium hydroxide), immediately mixed by inversion and incubated at room temperature for 1 minutes and the intensity of developed color was measured by spectroscopy at 436 nm. The amount of ammonia released was determined from an ammonium standard curve at 0.7 to 2.8 mM concentration range. (Ammonia standard solutions were prepared by dissolving ammonium sulfate in ultrapure water.) The enzyme activity was expressed as international unit (IU) defined as the amount of enzyme needed to liberate one micromole of ammonium per minute at pH 8.6 at 37 °C. All the measurements were done in triplicates.

## Results

###  Design of novel L-asparaginase

 To design a new L-asparaginase based on the sequence of the commercially available enzyme from *E. coli*, all reported mutations which are known to have beneficial structural and functional effects on the enzyme were extracted from the literature (Table 1). The list of mutations are as follows: N248A, R195A, K196A, H197A, N178P, N24A, R195S, Y250L, Y176S, W66Y, Y176F, K288S, K139A, V27T, L207A, Q59L, N24S, L1G, K107L, S241C, R269F. (The last four are equivalent to L23G/K129L/S263C/R291F mutations based on the numbering in reference Mahboobi et al, 2017.^33^ Residue numbering in the structure of L-asparaginase, which was utilized in this study for FoldX calculations is according to Verma et al, 2014, ignoring the first 22 residues belonging to the signal peptide.^34^

**Table 1:** Mutations and their effects on E. coli L-asparaginase

**Mutation **	**Results Achieved **	**References**
N248A	0.2% glutaminase activity and 12% L-asparaginase activity	^35^
R195A/K196A/H197A	Reduction in antigenicity	^36^
N178P	Retention of 90% L-asparaginase activity at 50 °C (wild-type 71%)	^37^
N24A/R195S	50% glutaminase activity and ∼ = 100% L-asparaginase activity	^38^
N24A/Y250L	∼ = 0% glutaminase activity and ∼ = 72% L-asparaginase activity	
Y176S	Increase of Vmax/KM for L-aspartic acid beta-hydroxamate	^39^
W66Y	Induced significantly more apoptosis in lymphocytes from ALL patients	
Y176F	Glutaminase activity reduction and ∼ = 100% L-asparaginase activity	
Y176S	Glutaminase activity reduction and ∼ = 100% L-asparaginase activity	
K288S/Y176F	Glutaminase activity reduction and ∼ = 100% L-asparaginase activity	
K288S/Y176F	10-fold less immunogenic	
K139A	Retention of 65% L-asparaginase activity at 65 °C (wild-type 40%)	
L207A	Retention of 57% L-asparaginase activity at 65 °C (wild-type 40%)	
Y176F	Increase of Vmax/KM for L-aspartic acid beta-hydroxamate	^34^
Q59L	0% Glutaminase activity and 80% L-asparaginase activity	^40^
N24S	Improved thermal stability and proteases resistant	^41^
L1G/K107L/S241C/R269F	Non-toxic, more stability and longer half life	^33^
V27T	Glutaminase activity reduction and more stable	^42^
R195A/K196A/H197A	Reduction in antigenicity	^36^
N178P	Retention of 90% L-asparaginase activity at 50 °C (wild-type 71%)	^37^

 All 2 097 152 possible mutants of the enzyme, guided by combination of the above-mentioned mutations, were listed and among them, those with up to four mutations (7546 mutants) were selected. By modifying the experimental structure (PDB code: 6PA3) of *E. coli* L-asparaginase (i.e., mutating back to original residues (T89V/K162T)), a ligand-free L-asparaginase template was built, and used for generating model structures of all selected mutants. The generated models were evaluated in terms of stability using FoldX algorithm, which uses a force-field developed based on empirical effective energy function.^27,28^ The folding energy differences of *in silico* generated mutants relative to that of the wild-type enzyme were calculated and sorted as shown partially in Table 2.

**Table 2:** Partial list of in silico generated mutant structures of L-asparaginase and their folding energy differences with wild-type enzyme calculated using FoldX algorithm

**Mutant structure file**	**Mutations**	**∆Δ*****G***** (kcal mol**^-1^**)**
ASP_4049.pdb	N248A,S241C	-2.17356
ASP_4224.pdb	Y176F,S241C	-2.06854
ASP_5003.pdb	N24S,K107L,S241C	-2.05545
ASP_7201.pdb	N248A,N24A,S241C,V27T	-2.03916
ASP_4717.pdb	N248A,K207A,N24S,S241C	-2.03252
ASP_4641.pdb	N248A,N24A,N24S,S241C	-2.01755
ASP_4079.pdb	N248A,K196A,N24A,S241C	-2.01354
ASP_4961.pdb	K139A,K107L,S241C	-1.96704
ASP_4624.pdb	N24S,S241C	-1.94765
ASP_4427.pdb	N248A,K207A,S241C	-1.92994
ASP_4229.pdb	N248A,K196A,Y176F,S241C	-1.79534
ASP_4920.pdb	N248A,Y176S,K107L,S241C	-1.71816
ASP_4731.pdb	N248A,Q59L,N24S,S241C	-1.664

The list was sorted based on folding energy difference (∆∆G) and 13 top scoring models are shown as the representatives.

**Figure 1:**
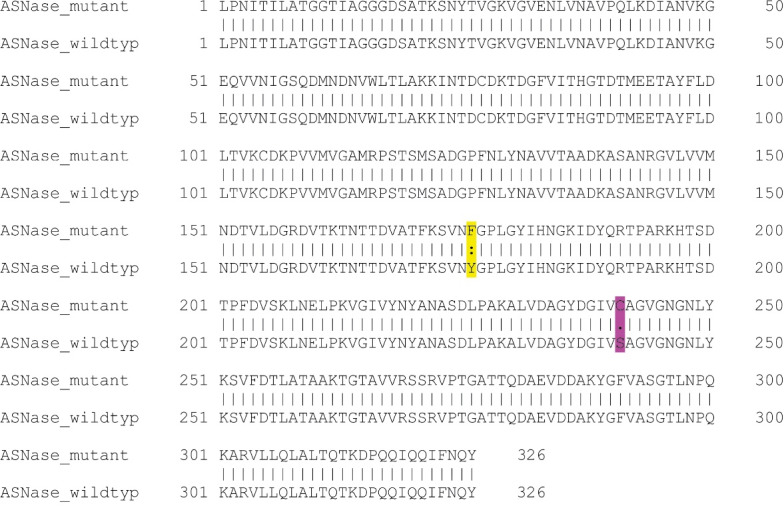
Pairwise sequence alignment of the selected Y176F/S241C double mutant L-asparaginase with the sequence of wildtype *E. coli* enzyme

 In this study, based on *in silico* stability assessment and reported functional and structural effects of various mutations, a novel double mutant L-asparaginase harboring Y176F and S241C mutations was selected for further experimental investigations. Pairwise sequence alignment of the selected double mutant L-asparaginase with the sequence of wildtype *E. coli* enzyme is shown in Figure 1.

**Figure 2:**
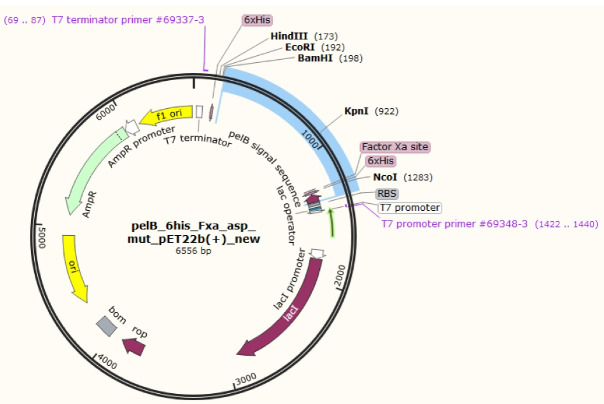
Expression vector where the gene for the designed Y176F/S241C double mutant L-asparaginase was inserted after pelB signal sequence, 6 × His tag and Factor Xa cleavage site

**Figure 3:**
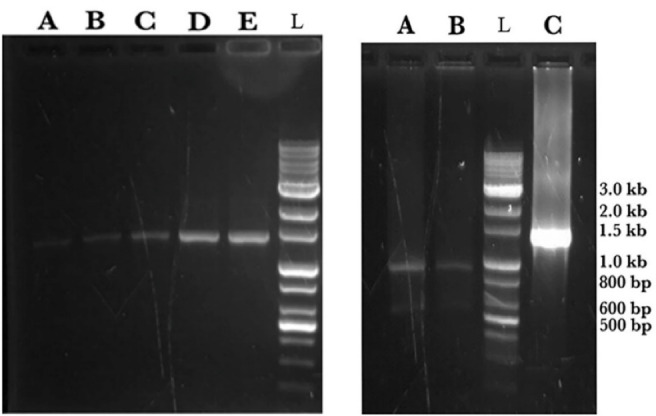
Agarose gel electrophoresis analysis on ASNase coding DNA plus flanking segments amplified in a PCR reaction using T7 promoter and T7 terminator primers. Left panel; The DNA bands at 1372 bp in lanes A, B, C, D and E are samples from PCR reaction loaded at different quantities and electrophoresed on1% gel. Right panel; Two DNA bands at 521 and 853 bp in lanes A and B are fragments from digestion of PCR product using KpnI restriction enzyme. Lane C is a sample from PCR reaction. In both panels the lane with DNA ladder is indicated by L.

 The gene sequence of the designed mutant ASNase was inserted virtually into pET22b( + ) vector and the designed construct shown in Figure 2 was ordered. Upon receiving, the designed plasmid was amplified and used in PCR reaction to amplify a DNA segment (1372 bp) encompassing L-asparaginase gene and flanking T7 promoter and T7 terminator sequences. The amplified PCR product was subjected to digestion using the KpnI restriction enzyme to yield two DNA fragments with the size of 521 and 853 bp (Figure 3).

###  Expression and detection of recombinant ASNase enzyme

**Figure 4:**
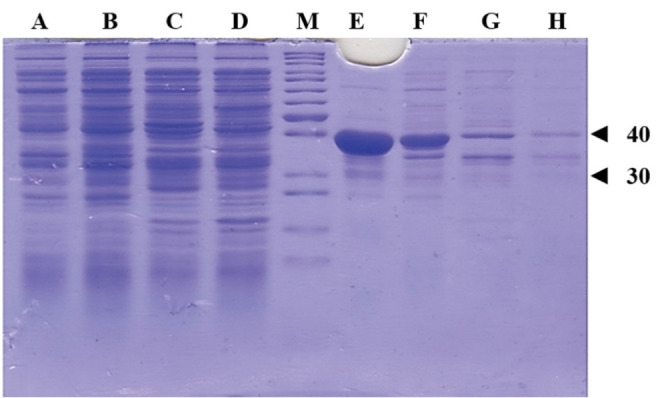
SDS-PAGE analysis of the produced ASNase protein from deferent E. coli strains. The bands around ~40 kDa represents the ASNase. Lanes A and B are soluble fractions of E. coli pLysS grown in 37 and 20 °C, respectively. Lanes C and D are soluble fractions of E. coli Origami grown in 37 and 20 °C, respectively. Lanes E and F are insoluble fractions of E. coli pLysS grown in 37 and 20 °C, respectively. Lanes G and H are insoluble fractions of E. coli Origami grown in 37 and 20 °C, respectively. Lane M is protein weight marker and bands corresponding to proteins with 30 and 40 kDa are indicated with triangles.

**Figure 5:**
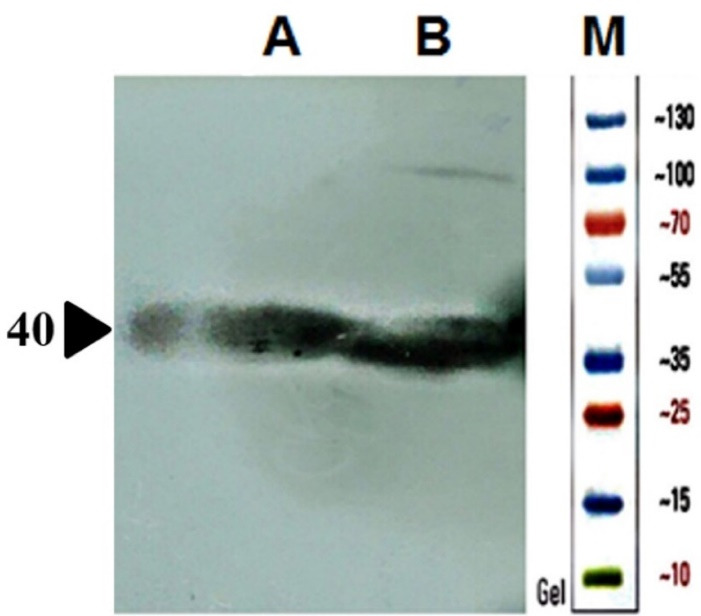
SDS-PAGE analysis of the produced ASNase protein in different *E. coli* strains transformed by a plasmid harboring ASNase coding gene. The bands around 40 kDa are ASNase. Lanes A and B are respectively samples from soluble fractions of *E. coli* pLysS and Origami strains expressing ASNase.

 Mutant ASNase protein was expressed by transforming the constructed plasmid shown in Figure 2 into two different *E. coli* strains (Origami and pLysS). The construct was designed in such a way that the protein sequence of mutant ASNase is preceded by pelB signal sequence, 6 × His tag, and Factor Xa cleavage site. The band for protein of interest was appeared close to 40 kDa (Figure 4). To confirm the expression of mutant ASNase protein, western blotting experiment was carried out. The protein contains 6 × His-tag, which could be identified by an anti-6 × His antibody in an immunoblotting experiment. The result of western blotting is shown in Figure 5, where the protein band at about ~ 40 kDa represent ASNase protein.

###  Detection of ASNase activity

 The activity of recombinant engineered ASNase was assayed using the spectroscopic method described in Materials and Methods section. Table 3 illustrate the results of enzyme activity assay using the produced enzyme present in soluble fractions of cell lysates prepared from bacterial cells (Origami and pLysS) expressing the designed ASNase, and a commercially available *E. coli* L-asparaginase (Bionase^®^).

**Table 3:** L-asparaginase activity of expressed enzyme in soluble fraction of transformed *E. coli*

**Sample**	**Contents**	**L-asparaginase activity (IU ± SD)**	**L-glutaminase activity (IU ± SD)**
A	Soluble fraction of cell lysate prepared from transformed *E.coli* BL21 pLysS	25.8 ± 2.5	2.7 ± 1.1
B	Soluble fraction of cell lysate prepared from transformed *E.coli* Origami	95.4 ± 7.5	13.3 ± 1.0
C	Commercial enzyme 0.5 mg/mL (positive control)	83.9 ± 11.0	53.3 ± 1.9

L-asparaginase activity (IU) values are for 0.1 mL of crude soluble fraction of cell lysate or 0.1 mL of pure commercial enzyme prepared at 0.5 mg/mL concentration.

## Discussion

 Acute lymphoblastic leukemia, caused by aberrant growth of undifferentiated lymphoid cells, can affect bone marrow, blood, and extramedullary locations.^43^ Treatment with L-asparaginase has become an essential component of therapy for patients (particularly children) with ALL. L-asparaginase is now commonly used in conjunction with other chemotherapeutic drugs during ALL therapy.^44^ Despite its numerous efficiencies, L-asparaginase application is associated with several restrictions. Thrombotic complications, hemorrhage and allergic reactions along with other side effects are among major limitations.^10,11,13^ The early inactivation of *E. coli* L-asparaginase (EcA) by circulating antibodies and the associated shortened *in vivo* half-life of the enzyme remain a key concern in ALL treatment, despite great advances in clinical effectiveness.^34^ Therefore, efficient L-asparaginase from different sources or even innovative bio-superior enzymes are needed.^2^ Bio-superiors will benefit patients since they may provide higher safety, improved efficacy, lower doses, and more economic therapy.^16^

 The ultimate goal is to produce a recombinant ASNase enzyme with high effectiveness and low L-glutaminase activity for the development of a new recombinant efficient enzyme with improved therapeutic properties. To anticipate the optimum protein engineering method, a variety of strategies may be applied by manipulating ASNase sequence using computational, experimental, and structural analysis.

 In this study, pursuing the ultimate goal, twenty-one significant known mutations in the sequence of *E. coli* ASNase reported in the literature were identified and then were utilized to build a list of L-asparaginase mutants that included all potential combinations of up to four amino acid changes in the sequence of *E. coli* ASNase guided by the known mutations. All 7546 suggested mutants have their models created and their stabilities calculated by FoldX empirical force field. This method rapidly evaluates the effect of mutations on the stability, folding and dynamics of a macromolecule based on its high-resolution 3D structure. The higher folding stability means better thermal tolerability, which is considered advantage for an enzyme.^13,45,46^ The results of folding stability calculations using FoldX algorithm indicated N248A/S241C double mutant ASNase as the most energetically stable enzyme. The same calculations showed that Y176F/S241C double mutation of L-asparaginase may also considerably improve its folding stability relative to the wildtype. This is in line with the experimental results reported in the literature, where Y176F mutation has led to some desirable effects.^34^ The experimentally observed improvement of ASNase with Y176F mutation could be partially explained by its higher stability and folding potential energy. On the other hand, it has been demonstrated experimentally that N248A mutation decreases ASNase activity.^35^ Therefore, to be on the safe side, Y176F/S241C was preferred over N248A/S241C mutant for further investigation. There is no experimental data for combined effect of Y176F/S241C double mutation on *E. coli* ASNase. However, as stated before, all mutations analyzed in this work have already been investigated solo or in combination with others. The results obtained here are in agreement with those reported in literature as discussed next. It has been shown that, in lymphocytes generated from ALL patients, Y176F mutant ASNase caused considerably higher apoptosis with considerably low glutaminase activity.^39^ This also was shown for other mutants with amino acid variation at position 176, such as Y176S single-, and K288S/Y176F double-mutated ASNases. It is worth mentioning that the K288S/Y176F double mutation reduces EcA’s antigenicity, and mice inoculated with K288S/Y176F variation had 10-fold lower IgG and IgM titers than mice immunized with wild-type EcA.^39^ According to another study, Y176F mutant showed an increase of enzyme efficiency as indicated by an increase in V_max_/K_M_ for L-aspartic acid beta-hydroxamate.^34^

 In an *in silico* study, S241C mutation was predicted to benefit ASNase functionality.^33^ The same study revealed that S241C mutation along with L1G, K107L and R269F substitutions not only stabilize protein but also make it non-toxic, with longer half-life and low antigenicity.^33^ Sequence analysis of the ASNase designed in the current study using ToxinPred method showed that the introduced Y176F/S241C mutations did not affect the toxicity of the enzyme. A sequence corresponding to positions 125 to 134 (VKCDKPVVMV) is the only segment common to both of the designed and wild type *E. coli* ASNases which is predicted to be toxic. From antigenicity point of view, Y176F/S241C double mutant ASNase was predicted to be less immunogenic than the wildtype ASNase according to AlgPred 2.0 algorithm.

 Based on the results discussed above, a novel double mutant enzyme with potentially improved enzymatic properties was proposed and then the process of production of the proposed mutant were performed using recombinant protein technology. The gene sequence for the chosen L-asparaginase mutant was created using the gene sequence of the wild type enzyme from *E. coli* and inserted into the pET22b ( + ) expression vector. The construct was designed to include a 6 × HIS tag, an appropriate signal sequence, and a cleavage site for factor Xa protease. The final genetic construct was ordered for synthesis through Pishgam Biotech Co in Tehran, Iran. The mutant ASNase protein was expressed in different *E. coli* strains using the generated plasmid and then analyzed using SDS-PAGE, and western blotting experiments. The protein bands at approximately ∼ 40 kDa represent the expressed Y176F/S241C double mutant ASNase in soluble and insoluble fractions of two different *E. coli* strains (pLysS and Origami) transformed by the used construct. The results also indicate that the expressed enzyme in the transformed *E. coli* pLysS strain goes mainly into insoluble fraction. In contrast, the levels of ASNase protein are almost qualitatively equal in both soluble and insoluble fractions of *E. coli* Origami strain.

 L-asparaginase catalyzes the conversion of L-asparagine to L-aspartic acid and ammonia, which subsequently interacts with the Nessler reagent to produce an orange-colored product. The activity of the produced novel recombinant Y176F/S241C double mutant ASNase was detected by Nessler reaction using samples prepared from soluble fractions of *E. coli* cells (pLysS and Origami) expressing the designed enzyme. The results indicated that the introduced mutations, i.e., Y176F/S241C, do not adversely affect the folding and catalytic activity of *E. coli* ASNase which is in agreement with previous reports. The total amount of expressed double-mutated ASNase in *E. coli* pLysS seems to be more than that in *E. coli* Origami. But most of it goes to insoluble fraction. The total protein contents of the soluble fractions prepared from both transformed *E. coli* strains used in this study for the expression of the designed ASNase were almost the same (~40 µg/mL for 100 times diluted sample). However, L-asparaginase activity of soluble fraction prepared from ASNase expressing Origami B(DE3) cells were almost 4 times higher than that of samples prepared from *E. coli* pLysS (Table 3). The higher L-asparaginase activity may be due to higher level of ASNase present in soluble fraction prepared form transformed Origami cells compared to transformed pLysS cells. Considering that ASNase has a disulfate bond between C77 and C105 residues, the observed higher L-asparaginase activity in soluble fraction of ASNase expressing Origami B(DE3) cells may also be due to relatively more proper folding of the enzyme in reducing cytoplasmic environment of Origami B(DE3) cells compared to pLysS cells, which in turn may be the reason for relatively higher level of enzyme in soluble fraction of Origami than pLysS. In contrast, the amount of the enzyme which goes into insoluble fraction of Origami B(DE3) cells is much less than that in pLysS cells as shown in Figure 4. It is noteworthy to compare the enzymatic activities (i.e., L-asparaginase and L-glutaminase activities) of the soluble fraction prepared from ASNase expressing Origami cells to the activities of commercial product. According to the results shown in Table 3, at the concentrations of the enzymes which leads to almost equal L-asparaginase activity (95.4 and 83.9 IU for novel enzyme and commercial enzyme, respectively), the glutaminase activity for the novel ASNase is much lower (13.3 and 53.3 IU for novel enzyme and commercial enzyme, respectively), suggesting a more suitable activity profile for the novel enzyme. According to the preliminary results presented in this work, the proposed designed ASNase appears to be a promising alternative to wild-type enzyme with therapeutic potentials. However, there are shortcomings which need to be dealt with, such as low expression level in soluble form, and address issues regarding protein purification and activity and kinetics measurements. Further studies are required to optimize soluble expression of the designed ASNase, for instance, by changing the expression vector, using different tags and testing various growth conditions. Nevertheless, the current work lays the groundwork for future experimental and clinical studies towards introduction of novel ASNase bio-better.

## Conclusion

 According to the presented computational study, Y176F/S241C double mutations may significantly increase *E. coli* L-asparaginase folding stability. Based on available experimental data from the literature, these mutations can improve enzymatic properties by increasing activity and stability, as well as reducing L-glutaminase activity and immunogenicity. Assessment of L- asparaginase activity of soluble fractions from *E. coli* strains expressing the designed novel ASNase indicated that the produced enzyme is active with much lower L-glutaminase activity compared to commercial ASNase (wild type enzyme from *E. coli*). Based on the findings of this study, we believe the data may indicate that the identified novel enzyme has potential for pharmaceutical and industrial applications.

## Acknowledgments

 Authors would like to thank Research Office and Biotechnology Research Center of Tabriz University of Medical Sciences for providing financial support and laboratory facilities.

## Competing Interests

 The authors declare no conflict of interest.

## Ethical Approval

 Not applicable.
